# Effect of the Key Mixture Parameters on Shrinkage of Reactive Powder Concrete

**DOI:** 10.1155/2014/426921

**Published:** 2014-06-23

**Authors:** Shamsad Ahmad, Ahmed Zubair, Mohammed Maslehuddin

**Affiliations:** ^1^Civil and Environmental Engineering Department, King Fahd University of Petroleum and Minerals, Dhahran 31261, Saudi Arabia; ^2^Research Institute, King Fahd University of Petroleum and Minerals, Dhahran 31261, Saudi Arabia

## Abstract

Reactive powder concrete (RPC) mixtures are reported to have excellent mechanical and durability characteristics. However, such concrete mixtures having high amount of cementitious materials may have high early shrinkage causing cracking of concrete. In the present work, an attempt has been made to study the simultaneous effects of three key mixture parameters on shrinkage of the RPC mixtures. Considering three different levels of the three key mixture factors, a total of 27 mixtures of RPC were prepared according to 3^3^ factorial experiment design. The specimens belonging to all 27 mixtures were monitored for shrinkage at different ages over a total period of 90 days. The test results were plotted to observe the variation of shrinkage with time and to see the effects of the key mixture factors. The experimental data pertaining to 90-day shrinkage were used to conduct analysis of variance to identify significance of each factor and to obtain an empirical equation correlating the shrinkage of RPC with the three key mixture factors. The rate of development of shrinkage at early ages was higher. The water to binder ratio was found to be the most prominent factor followed by cement content with the least effect of silica fume content.

## 1. Introduction

Reactive powder concrete (RPC), also known as ultrahigh performance concrete (UHPC), is produced by mixing water, Portland cement, silica fume, fine quartz sand, quartz powder, superplasticizer, and steel fibers. The microstructure of RPC is made highly dense as it is prepared with a very low water to binder ratio (0.15 to 0.20), high cement content (800 to 1100 kg/m^3^), high silica fume content (20 to 25% of the weight of cement), well-graded fine sand and quartz powder (without using coarse aggregate), and steel fibers (about 6% by volume of RPC) [1]. The dense microstructure and presence of steel fibers in RPC mixtures provide superior mechanical properties (high strength, ductility, and toughness) and durability characteristics compared to conventional high performance concretes [2, 3]. However, due to use of high amounts of cement and silica fume at a very low water to binder ratio in preparing the RPC mixtures, the risk of high autogenous shrinkage at early ages cannot be ruled out.

In normal concrete, total shrinkage is taken as the sum of drying shrinkage (due to loss of moisture through evaporation) and autogenous shrinkage (due to consumption of water for continued hydration of cementitious materials after initial setting through a process termed as self-desiccation). The autogenous shrinkage in normal concrete is found to be very small particularly when the water to cement ratio is above 0.42. However, due to low water to binder ratio and high silica fume content used for producing the RPC mixtures, high self-desiccation takes place causing autogenous shrinkage of high order of magnitude [1, 4]. Self-desiccation of paste in concrete, which takes place due to continued hydration of cementitious materials at very low water to binder ratio and high silica fume content, results in decline in its autogenous relative humidity which leads to the autogenous shrinkage. Initially, the internal relative humidity is high but, with the progress of self-desiccation, the internal relative humidity decreases at faster rate causing autogenous shrinkage at high rate. Loukili et al. [1] have reported 1-day and 10-day autogenous shrinkage as 45% and 95% of the final autogenous shrinkage, respectively, for RPC with a water to binder ratio of 0.20 and silica fume content of 24% by the mass of cement. This indicates the fast occurrence of autogenous shrinkage to a level near to the ultimate value at an early stage; however, with passage of time, the rate of decrease in the internal relative humidity falls due to decrease in internal temperature and moisture, thereby decreasing the rate of self-desiccation and therefore the rate of autogenous shrinkage leading towards steady-state condition [1, 4–6]. Tazawa and Miyazawa [7] have reported that, at very low water to binder ratio (around 0.17), the autogenous shrinkage of concrete could be nearly the same as drying shrinkage.

The risk of autogenous shrinkage increases with decrease in the water to binder ratio and with increase or decrease of the amount of the mineral admixtures depending on their types and chemical and physical properties [4, 8–10]. Jiang et al. [8] have reported a sharp decrease in the autogenous relative humidity at lower water to binder ratio and increased amount of the mineral admixtures increasing the chances of more autogenous shrinkage. However, the lowering of water to binder ratio increased the autogenous shrinkage more than the increasing of the dosage of mineral admixtures. Unlike the autogenous shrinkage, the drying shrinkage in RPC decreases with decrease in the water to binder ratio [10, 11]. For concrete with low water to binder ratio, both fly ash and slag decrease the drying shrinkage while silica fume increases the drying shrinkage depending on other factors such as curing time, measurement method, curing type, cement replacement ratios, and so forth. On the other hand, while the addition of fly ash reduces the autogenous shrinkage, the addition of slag or silica fume increases the autogenous shrinkage [10]. While the use of a high dosage of superplasticizer increases the drying shrinkage [11], the expansive and shrinkage reducing admixtures can decrease the shrinkage in RPC mixtures significantly [12, 13]. The use of superabsorbent polymers has been reported to be effective in reducing the autogenous shrinkage [14]. The rice husk ash is also found to be useful in mitigating the autogenous shrinkage in the RPC mixtures [15]. The effect of the steel fibers in RPC mixtures is to reduce the shrinkage by restraining effect offered by the fibers [1, 16]. Garas et al. [17] have reported a reduction in free shrinkage by about 50% and autogenous shrinkage by about 42% by adding 2% of steel fibers (by volume) to RPC mixture.

While the effect of the various parameters (water to binder ratio, mineral admixtures, superplasticizer, shrinkage reducing admixtures, steel fibers, etc.) on the shrinkage of RPC mixtures is reported individually in the literature, the simultaneous effects of the key mixture parameters such as water to binder ratio, cement content, and silica fume content on shrinkage are rarely addressed. If the shrinkage is quantitatively correlated to the key mixture parameters, the mixture proportions can be optimized to keep the shrinkage of RPC mixtures within the permissible limits. In the present work, an attempt has been made to study the simultaneous effects of three key mixture parameters (water to binder ratio, cement content, and silica fume content) on shrinkage of the RPC mixtures. Considering three different levels of the three key mixture factors, a total of 27 mixtures of RPC were prepared according to 3^3^ factorial experiment design. The specimens belonging to all 27 mixtures were monitored for shrinkage at different ages over a total period of 90 days. The test results were plotted to observe the variation of shrinkage with time and to see the effects of the key mixture factors. The experimental data pertaining to 90-day shrinkage (ultimate shrinkage) were used to conduct analysis of variance (ANOVA) to identify significance of each factor and to obtain an empirical equation correlating the shrinkage of RPC with the three key mixture factors.

## 2. Experimental Program

### 2.1. Materials

#### 2.1.1. Cement and Silica Fume

Type I cement (ordinary Portland cement) conforming to ASTM C 150 [18] with a specific gravity of 3.15 and chemical composition, as shown in Table 1, was used in all the mixtures of RPC. The chemical composition of the silica fume used is also shown in Table 1.

#### 2.1.2. Fine Dune Sand

Fine dune sand obtained from the deserts of Saudi Arabia, with water absorption of 0.5% and specific gravity of 2.53, was used as aggregate. The natural grading of sand used in all the mixtures is shown in Table 2.

#### 2.1.3. Superplasticizer and Steel Fibers

A liquid superplasticizer (commercial name: Glenium 51) was used to obtain the desired flow. Glenium 51 is a polycarboxylic ether (PCE) based superplasticizer which does not contain chlorides and complies with ASTM C494 [19] Types A and F. The specific gravity of Glenium 51 was 1.095 with 65% water content by weight. Varying dosage of this superplasticizer was used to obtain a flow of 200 ± 20 mm for all the mixtures using ASTM C1437 (standard test method for measuring flow of hydraulic cement mortar) [20]. Steel fibres of 0.22 mm diameter and 13 mm length with tensile strength greater than 2850 MPa were used in all the mixtures.

### 2.2. RPC Mixtures

Three key mixture parameters, namely, water/binder ratio, cement content, and silica fume content, were considered with their three levels of variation for studying the effect of these mixture parameters on the shrinkage of a total of 27 RPC mixtures as per 3^3^ factorial experiment design. The three levels of each parameter, selected within their practical ranges for producing RPC, are given below:

 water/binder (w/b) ratio:   0.15, 0.175, and 0.20;
 cement content (kg/m^3^): 1000, 1100, and 1200;
 silica fume content (% of cement): 15, 20, and 25.

Steel fiber content of 157 kg/m^3^ was used in all the mixtures. Absolute volume method was used to design the mixtures. The weights of constituents determined for producing one cubic meter of each of the RPC mixtures are presented in Table 3.

### 2.3. Preparation of Test Specimens

Since RPC is composed of very fine materials; special attention was needed for mixing. The procedure recommended in the literature [21–23] for mixing of RPC was followed. After mixing, the RPC was poured into the molds and consolidated using a vibrating table. A total of 81prisms of size 25 × 25 × 275 mm (27 mixtures × 3 replicates) were cast for monitoring the drying shrinkage. The specimens were first cured in a water tank for 3 days after casting. After 3 days of water-curing, specimens were taken out of the tank and the surfaces were allowed to dry before starting the shrinkage monitoring in the laboratory conditions (50% relative humidity and 25°C temperature).

### 2.4. Monitoring Total Shrinkage of the Specimens

**Figure 1:**
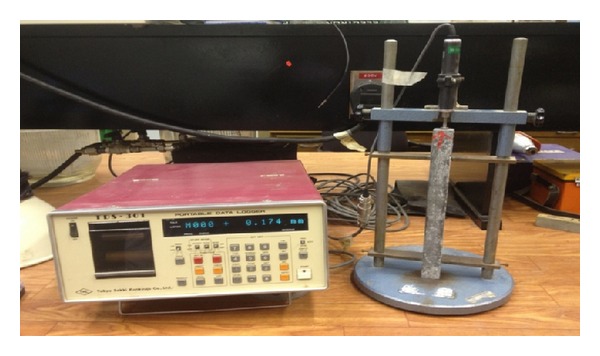
Setup for measuring total shrinkage.

Total shrinkage of the specimens belonging to all 27 mixtures of RPC was measured at 3, 7, 14, 21, 28, 42, 56, and 90 days after casting according to ASTM C157 [24]. For each mixture, total shrinkage was measured at a time on a set of three similar specimens and average of three measured values of total shrinkage was reported. A setup consisting of a stand fitted with a LVDT connected to a data logger was used, as shown in Figure 1.

## 3. Results and Discussion

### 3.1. Effect of Age on Shrinkage

**Figure 2:**
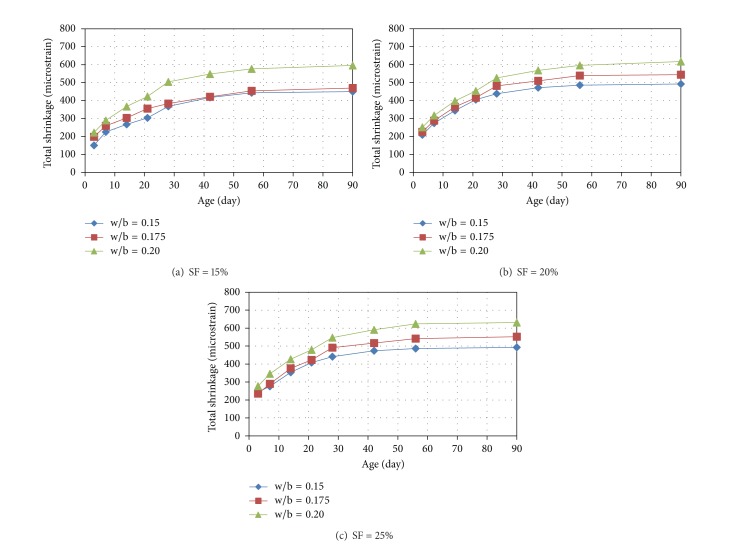
Variation of total shrinkage of RPC mixtures (having cement content of 1000 kg/m^3^) with age.

**Figure 3:**
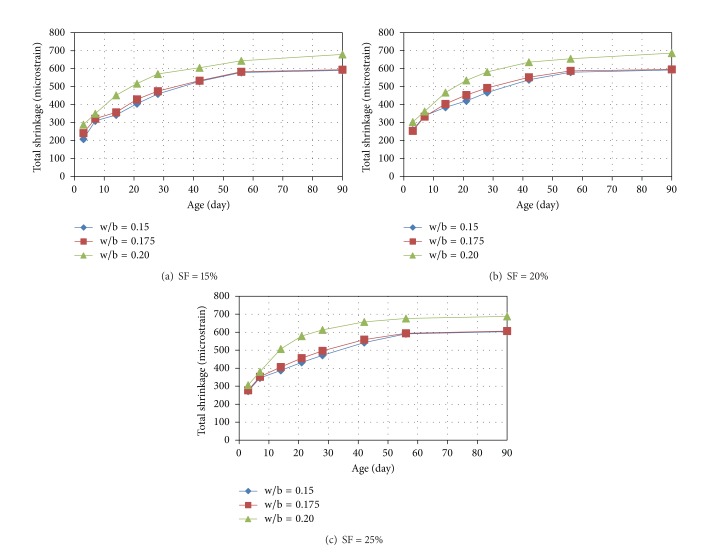
Variation of total shrinkage of RPC mixtures (having cement content of 1100 kg/m^3^) with age.

**Figure 4:**
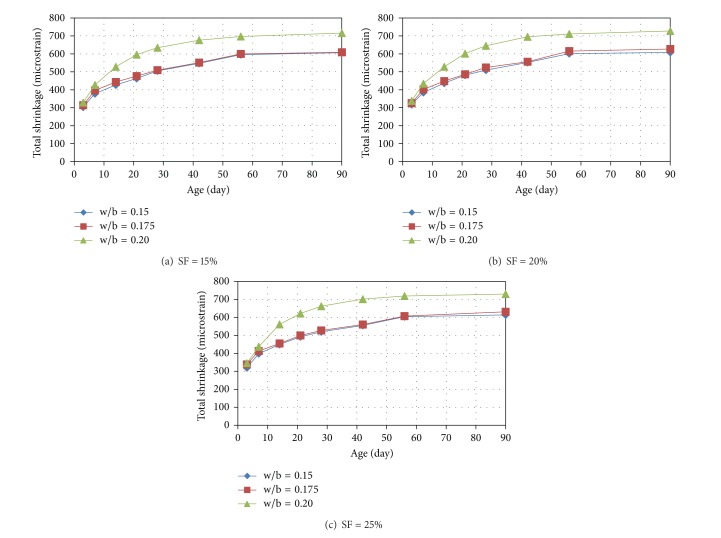
Variation of total shrinkage of RPC mixtures (having cement content of 1200 kg/m^3^) with age.

The plots showing the variation of the total shrinkage of the mixtures of RPC with age are shown in Figures 2, 3, and 4. It can be seen from Figures 2 through 4 that the rate of development of shrinkage is higher at the early stages and almost a steady-state condition is observed in case of all the mixtures at the age of 90 days. The shrinkage at 3, 7, 14, 21, 28, 42, and 56 days were found in the ranges of 33 to 54, 48 to 65, 58 to 77, 67 to 85, 78 to 91, 89 to 96, and 95 to 99% of the 90-day shrinkage (i.e., typically ultimate shrinkage), respectively. It can be noted that more than one-third, one-half, and two-thirds of ultimate shrinkage occurred at the early ages of 3, 7, and 14 days.

### 3.2. Effects of Mixture Parameters on Shrinkage

From the observation of Figures 2 through 4, it can be seen that with increase in the w/b ratio the total shrinkage increases. However, the increase in the total shrinkage with increase of w/b ratio from 0.15 to 0.175 is almost negligible particularly at cement content above 1000 kg/m^3^. This is because of the fact that, at an intended w/b ratio of 0.15, the required amount of superplasticizer is very high resulting in an effective w/b ratio almost similar to that for the intended w/b ratio of 0.175, as evident from the values of effective w/b ratios presented in the last column of Table 3. There is significant increase in the shrinkage when w/b ratio was increased from 0.175 to 0.20 because there is significant difference between the corresponding effective w/b ratios also due to relatively lower amount of superplasticizer required at the intended w/b ratios of 0.175 and 0.2, particularly for the cement content of more than 1000 kg/m^3^.

A significant increase in the shrinkage with increase in cement content can be observed from Figures 2 through 4. It can be further noted that the increase in the shrinkage is more significant when cement content was increased from 1000 to 1100 kg/m^3^ as compared to increase in shrinkage due to increase in cement content from 1100 to 1200 kg/m^3^. The reason behind insignificant increase in the shrinkage when cement content in the present study was increased from 1100 to 1200 kg/m^3^ may be attributed to the following fact. Although, with increase in the cement content, self-desiccation increases causing more shrinkage, beyond certain point, the self-desiccation helps in hydrating the cement partially and the additional cement mostly remains unhydrated limiting the effect of increase in the cement content on the shrinkage. Further, it may be noted that the effect of cement content on shrinkage is higher at lower w/b ratio and lower silica fume content.

The effect of increase in the silica fume content in the range of 15 to 25% was found to be marginal at cement content of 1000 kg/m^3^. However, the effect of increase in the silica fume content was insignificant at cement content above 1000 kg/m^3^.

### 3.3. Statistical Analysis of the 90-Day Shrinkage Test Results

In order to compare the significance of each of the three key mixture factors on shrinkage, the analysis of variance (ANOVA) was conducted using 90-day shrinkage (ultimate shrinkage) data. The ANOVA results (*F*-ratios), as summarized in Table 4, indicate that the w/b ratio has most significant effect followed by cement content with the least effect of silica fume content. The variation of the shrinkage with the selected key mixture parameters indicates the possibility of optimizing the mixtures of RPC if a regression equation correlating shrinkage with the mixture parameters is obtained utilizing the experimental data generated through the present work.

The regression equation relating the 90-day shrinkage to the w/b ratio, cement content, and silica fume content is given as follows:

(1) Sh=2401  W+0.529  C+0.247  S−445              [R2=0.99],

where *S*_*h*_ is 90-day shrinkage (microstrain), *W* is w/b ratio (by mass), *C* is cement content (kg/m^3^), *S* is silica fume content Kg/m^3^.

The above regression equation indicates that an increase in the paste volume due to higher cement and mineral additive content is one of the main reasons of higher ultimate shrinkage for RPC. This is in agreement with the fact that more paste (i.e., less aggregate) in concrete increases the shrinkage [25]. The equation obtained for the ultimate shrinkage can be used to optimize the levels of w/b ratio, cement content, and silica fume content (within their ranges of variations considered in this study) for keeping the ultimate shrinkage at a required maximum permissible limit.

## 4. Conclusions

Based on the findings of the present study, the following conclusions can be made.

- The shrinkage in all the RPC mixtures occurred at high rates at early stages and almost reached the steady-state condition after an exposure period of 90 days. More than one-third, one-half, and two-thirds of the ultimate shrinkage occurred at the early ages of 3, 7, and 14 days.
- The increase in the total shrinkage with increase of w/b ratio from 0.15 to 0.175 is almost negligible particularly at cement content above 1000 kg/m^3^ because the effective w/b ratios corresponding to the intended w/b ratios of 0.15 and 0.175 were almost similar due to high requirement of superplasticizer at a w/b ratio of 0.15.
- Increase in the shrinkage was more significant when cement content was increased from 1000 to 1100 kg/m^3^ as compared to increase in shrinkage due to increase in cement content from 1100 to 1200 kg/m^3^ due to the fact that the self-desiccation is not proportional to the cement content beyond a certain limit.
- All the three key mixture parameters were found to increase the shrinkage with increase in their levels because of the increase in the paste content. The statistical analysis of the experimental data using ANOVA indicated that the w/b ratio affected shrinkage most significantly followed by the cement content with the least effect of the silica fume content.
- The regression equation obtained for the ultimate shrinkage of the RPC mixtures can be utilized to optimize the levels of w/b ratio, cement content, and silica fume content (within their ranges of variations considered in this study) for keeping the ultimate shrinkage at a required maximum permissible limit.

## Figures and Tables

**Table 1:** Chemical composition of cement and silica fume.

Constituent	Cement (%)	Silica fume (%)
CaO	64.35	0.41
SiO_2_	22.0	86.75
Al_2_O_3_	5.64	0.41
Fe_2_O_3_	3.80	2.12
K_2_O	0.36	0.67
MgO	2.11	0.18
Na_2_O	0.19	0.17
Equivalent alkalis (Na_2_O + 0.658K_2_O)	0.33	0.62
SO_3_	2.10	0.73
Loss on ignition	0.70	3.35

**Table 2:** Dune sand grading.

Sieve opening, mm	% passing
4.75	100
2.4	100
1.2	100
0.6	96.2
0.3	61.4
0.15	21.9
0.075	1.0

**Table 3:** Weights of the constituents of 27 RPC mixtures (for 1 m^3^).

Mix ID	w/b ratio	Cement (kg)	Silica fume (%)	Silica fume (kg)	Water (kg)	Fiber (kg)	SP (%)	SP (kg)	Sand (kg)	Effective w/b ratio considering water in SP
M1	0.15	1000	15	150	173	157	3.55	41	977	0.183
M2	0.15	1000	20	200	180	157	3.55	43	898	0.182
M3	0.15	1000	25	250	188	157	3.55	44	818	0.182
M4	0.15	1100	15	165	190	157	3.55	45	827	0.182
M5	0.15	1100	20	220	198	157	3.55	47	739	0.182
M6	0.15	1100	25	275	206	157	3.55	49	652	0.182
M7	0.15	1200	15	180	207	157	3.55	49	676	0.182
M8	0.15	1200	20	240	216	157	3.55	51	581	0.182
M9	0.15	1200	25	300	225	157	3.55	53	486	0.182
M10	0.175	1000	15	150	201	157	2	23	945	0.193
M11	0.175	1000	20	200	210	157	2	24	865	0.193
M12	0.175	1000	25	250	219	157	2	25	784	0.193
M13	0.175	1100	15	165	221	157	1.5	19	806	0.188
M14	0.175	1100	20	220	231	157	1.5	20	718	0.189
M15	0.175	1100	25	275	241	157	1.5	21	630	0.189
M16	0.175	1200	15	180	242	157	1.5	21	654	0.189
M17	0.175	1200	20	240	252	157	1.5	22	558	0.189
M18	0.175	1200	25	300	263	157	1.5	23	462	0.189
M19	0.20	1000	15	150	230	157	1.5	17	886	0.213
M20	0.20	1000	20	200	240	157	1.5	18	803	0.214
M21	0.20	1000	25	250	250	157	1.5	19	719	0.214
M22	0.20	1100	15	165	253	157	1	13	741	0.209
M23	0.20	1100	20	220	264	157	1	13	650	0.209
M24	0.20	1100	25	275	275	157	1	14	559	0.209
M25	0.20	1200	15	180	276	157	1	14	583	0.209
M26	0.20	1200	20	240	288	157	1	14	484	0.209
M27	0.20	1200	25	300	300	157	1	15	385	0.209

**Table 4:** Results of the ANOVA of the ultimate shrinkage (90-day) data.

Factor	Type	Levels	Values			
*W* (w/b ratio)	Fixed	3	0.150, 0.175, and 0.200			
*C* (cement content)	Fixed	3	1000, 1100, and 1200 Kg/m^3^			
*S* (SF content)	Fixed	3	15, 20, and 25% of cement content			

Analysis of variance (ANOVA), using adjusted SS for tests						

Source	Degree of freedom (DF)	Sum squares (SS)	Adjusted SS	Adjusted mean sum squares (MS)	*F*-ratio	Probability of no significance (*P*)

*W*	2	67027.4	67027.4	33513.7	358.68	0.000
*C*	2	62146.7	62146.7	31073.4	332.56	0.000
*S*	2	3029.3	3029.3	1514.6	16.21	0.002
*W*∗*C*	4	2353.7	2353.7	588.4	6.30	0.014
*C*∗*S*	4	2332.9	2332.9	583.2	6.24	0.014
*W*∗*S*	4	313.6	313.6	78.4	0.84	0.537
Error	8	747.5	747.5	93.4	—	—
Total	**26**	**137951.0**	—	—	—	—

*S* = 9.66623, *R*-Sq = 99.46%, *R*-Sq(adj) = 98.24%						
